# Involuntary admission in Norwegian adult psychiatric hospitals: a systematic review

**DOI:** 10.1186/s13033-018-0189-z

**Published:** 2018-03-22

**Authors:** Rolf Wynn

**Affiliations:** 1 Department of Clinical Medicine, Faculty of Health Sciences, UiT The Arctic University of Norway, 9037 Tromsø, Norway; 2 Division of Mental Health and Addictions, University Hospital of North Norway, Tromsø, Norway

**Keywords:** Psychiatry, Norway, Coercion, Involuntary admission, Civil commitment

## Abstract

**Background:**

It is an important objective of the psychiatric services to keep the use of involuntary procedures to a minimum, as the use of coercion involves clinical, ethical, and legal issues. It has been claimed that Norway has a relatively high rate of involuntary admissions. We reviewed the peer-reviewed literature on the use of involuntary admission in Norway, with the purpose of identifying the current state of knowledge and areas in need of further research.

**Methods:**

A systematic review following the PRISMA statement was conducted. We searched the electronic databases PsycInfo, PubMed, Web of Science, CINAHL, and Embase for studies relating to involuntary admission to Norwegian adult psychiatric hospitals published in the period 1 January 2001 to 8 August 2016. The database searches were supplemented with manual searches of relevant journals, reference lists, and websites.

**Results:**

Seventy-four articles were included and grouped into six categories based on their main topics: Patients’ experiences, satisfaction and perceived coercion (21 articles), the Referral and admission process (11 articles), Rates of admission (8 articles), Characteristics of the patients (17 articles), Staff attitudes (9 articles), and Outcomes (8 articles). Four of the included articles described intervention studies. Fifty-seven of the articles had a quantitative design, 16 had a qualitative design, and one a mixed-method design. There was a broad range of topics that were studied and considerable variation in study designs. The findings were largely in line with the international literature, but the particularities of Norwegian legislation and the Norwegian health services were reflected in the literature. The four intervention studies explored interventions for reducing rates of involuntary admission, such as modifying referring routines, improving patient information procedures, and increasing patients’ say in the admission process, and represent an important avenue for future research on involuntary admission in Norway.

**Conclusions:**

The review suggests that Norway has a relatively high rate of involuntary admissions. The identified studies represent a broad mix of topics and designs. Four intervention studies were identified. More studies with strong designs are needed to bring research on involuntary admission in Norway to a next level.

**Electronic supplementary material:**

The online version of this article (10.1186/s13033-018-0189-z) contains supplementary material, which is available to authorized users.

## Background

Involuntary admission to psychiatric hospital is problematic on several levels. The clinical benefit of admitting patients involuntarily has been disputed and there are legal and ethical issues related to coerced admission [1–8]. It is a basic principle that health services should be based on consent and that coercion should be reduced to the minimum possible [1–8]. Recent studies on the use of coercion in other countries, such as the EUNOMIA and the OPUS-trial, have highlighted these issues [5–8]. Other studies have suggested that the use of involuntary admission in Norway is relatively high compared to other Western countries [9, 10]. There is therefore considerable societal and political pressure to reduce coercion in Norwegian psychiatric services [11].

While there have been very recent changes to the Norwegian mental health legislation [12], when the search of the present study was performed, a patient could be admitted involuntarily to psychiatric hospital if he/she suffered from a severe mental disorder (i.e. a psychosis or equivalent) and if the admission was necessary for treatment and/or the patient was considered a danger to him/herself or to others [12]. An involuntary patient must be referred to psychiatric hospital by an independent doctor who personally has examined the patient and has found that the criteria are met. Voluntary treatment must have been tried or deemed futile. At admission (i.e. within 24 h), the patient is assessed by a specialist (psychiatrist or clinical psychologist), who decides whether the legal criteria are met. If the patient is admitted involuntarily, an independent review board, usually headed by a judge, will also assess the formalities of the admission. The patient may also launch a formal complaint and get a lawyer appointed free of charge to aid in the complaints process, which may also be appealed to the civil courts system.

Many of the issues relating to involuntary admission in Norway are common to other countries. However, a relatively high rate of involuntary admissions in Norway, the specifics of the Norwegian mental health legislation, and the organisation of the Norwegian mental health services, make it necessary to investigate the topic of involuntary admission within a Norwegian context. The literature on the use of involuntary admission in Norway has grown during the past decades, but an overview of the research, its methods and results, is lacking. By systematically reviewing the literature, this issue will be addressed and it will be possible to sum up the status on this field of research in Norway, including studies on patients’ experiences, the referral process, rates and characteristics of involuntary patients, staff attitudes and outcomes. The aim of the study is to identify findings that might help decision-makers and point to research areas and methodologies that can forward the field in Norway.

## Methods

### Search strategy

**Fig. 1:**
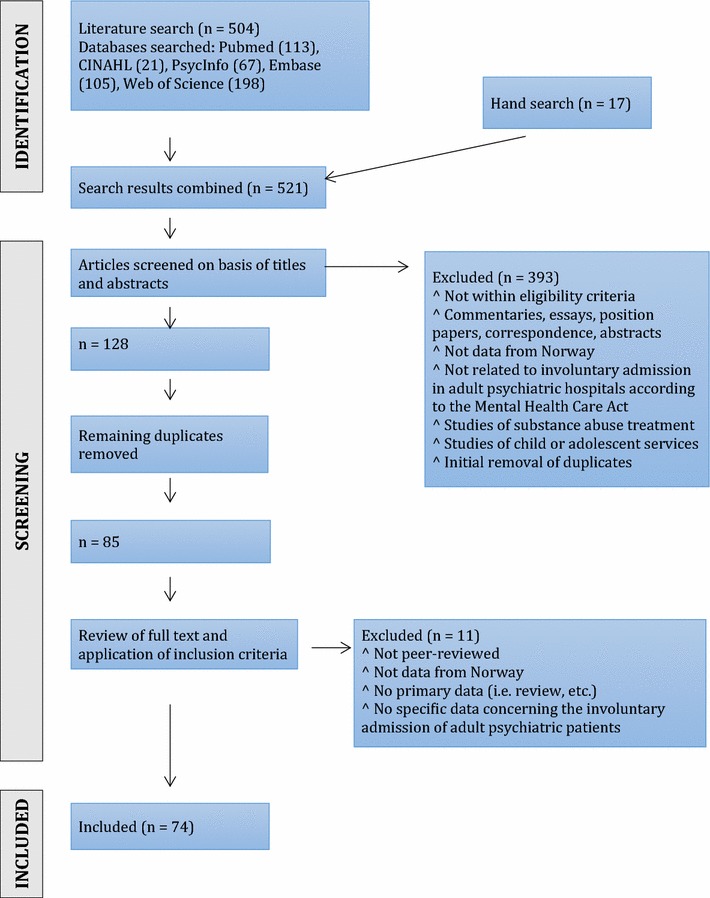
Process for selection of studies included in the review

This review follows the PRISMA Guidelines [13]. A systematic search of the literature in relevant electronic databases, including PsycInfo (Ovid), PubMed, Embase (Ovid), CINAHL (Ebsco), and Web of Science, was carried out in order to identify relevant articles. The databases were searched out on 8 August, 2016. The search consisted of different combinations of various terms related to involuntary admission in Norway, see Additional file 1 for more details. The search terms were in English, as Norwegian researchers in this field of study usually publish in English. There is a handful of scientific journals that occasionally publish relevant articles in Norwegian. However, these journals usually provide abstracts in English that are indexed in the databases that have been searched. Additional hand searches of relevant journals, including journals publishing in Norwegian, and searches on some Norwegian websites that might report on relevant studies, were also performed. See Fig. 1 for a flow chart describing the search process.

### Eligibility criteria

We considered for inclusion all articles published in peer-reviewed journals with data from Norway that involved the involuntary admission of patients to adult psychiatric hospitals subject to the Norwegian Mental Health Care Act [12]. We included articles published after 1 January 2001, as this was when a new Mental Health Care Act was implemented [12]. Articles that included primary data on referrals, service organisation, attitudes, and ethical issues were also considered for inclusion provided they focused on the topic of involuntary admission. We excluded articles that did not specifically mention involuntary admission or that examined coercion only as a general concept. We excluded studies involving only, admission to substance abuse treatment, and services for children, adolescents or the intellectually disabled, as these in Norway involve separate institutions/services and/or additional regulations that may impact coercive practices overall. We excluded studies without data from Norway, and studies that did not include primary empirical data (i.e. commentaries, position papers, reviews, etc.). Studies that dealt primarily with legal theory and that did not include service-related data were excluded as were studies published only as abstracts. We excluded surveys on rates of involuntary admission in Norway not published in peer-reviewed journals, but some such surveys have nevertheless been brought into the discussion.

## Results

### Results of search process

In total 504 articles were initially identified in the electronic search. Hand searches of relevant journals and reference lists of articles resulted in 17 additional articles. We screened the titles and abstracts of the publications for relevance, reducing the number to 128. Removing remaining duplicates further reduced the number to 85. These were read in full and considered against the eligibility criteria (described above), resulting in the 74 articles included in the review (see Fig. 1, Additional files 2, 3).

### Synthesis and analysis of data

We grouped the 74 included articles into six different categories according to their main topics. We devised the following categorisation: (1) Studies that examined patients’ experiences, patients’ satisfaction, or perceived coercion, (2) Studies that examined the referral or admission processes, (3) Studies that examined population-based rates of involuntary admission and factors that influenced rates, (4) Studies examining the characteristics of patients that had been involuntarily admitted, (5) Studies examining staff attitudes to involuntary admission, (6) Studies examining the outcomes of involuntary admissions. We condensed the central findings in each article in the different categories and we identified the common findings in the various categories (see Additional files 2, 3).

Seventy articles described observational studies and only four articles [14–17] described intervention studies. Fifty-seven of the studies drew on a quantitative methodology. One study [18] had a mixed-methods design. Sixteen studies had a qualitative design [19–34], i.e. including in-depth interviews [19, 20, 22–30, 34], focus group interviews [26, 30, 31], narrative text analysis [21, 23], and observation [19, 20, 30].

### Studies examining patients’ experiences, satisfaction, and perceived coercion

Twenty-one studies were identified [18–25, 32–43] that treated the topic of patients’ experiences, satisfaction and perceived coercion. Most of the studies involved some type of interview by either staff or researchers [18–20, 22–25, 30, 34, 35, 37, 39, 40]. In some of the interviews [18, 35, 37, 39, 40], rating scales were also used to measure results. Some studies sampled data by giving out questionnaires to patients to fill out [36, 38, 41–43]. Some studies drew on qualitative in-depth interviews [18–20, 22–25, 30] and/or focus group interviews [30, 34]. Other approaches, such as content analysis [33, 34] and participant observation [19, 20, 25, 30] were also used. Interestingly, among the 21 studies in this category, more than half had a qualitative design, one study [18] had mixed methods design, and only eight had a purely quantitative design.

### Main findings

Four studies [35–37, 39] drew on the concept of perceived coercion. One study [35] showed that involuntary patients had higher levels of perceived coercion, while another study [39] found that legal status did not significantly influence whether patients had high levels of perceived coercion or not. One study [37] showed that about half the involuntary patients felt coerced. Seclusion was a significant predictor of high levels of perceived coercion in one study [36]. Two studies [38, 41] showed that committed patients were the least satisfied with treatment, and another study [39] showed that accumulated coercive events and objective coercion negatively impacted satisfaction. One study [42] did not show any difference between the coerced and the non-coerced patients in terms of patients’ satisfaction. Some qualitative studies [19, 20, 25] pointed out that staff and patients had different perceptions of coercion and of triggers of dangerous situations. Staff that were close to patients were more likely to understand the patient’s perspectives. Some studies discussed the importance of providing services that the patients perceived of as helpful [32–34]. One study explored patients’ moral evaluation of coercion [30] and another study discussed the relationship between patients’ insight and coercion [23]. Two studies [22, 24] showed that some patients said they in retrospect understood the necessity of using coercion. Being committed predicted a high level of humiliation [40, 43] as did the conviction that ‘the admission was not right’ [18]. The involvement of police in fetching patients at home could represent trauma, debasement, and stigma [21].

### Studies examining the referral or admission processes

Eleven studies [26, 27, 44–52] examined the topic of the referral and/or the admission of involuntary patients to psychiatric hospitals. The studies were primarily based on the examination of medical records [44–48, 52] or on interviews with referring GPs [26, 49–51] or admitting specialists [27]. In this category, two of the 11 studies were qualitative [26, 27].

### Main findings

Out-of-hours clinics refer a higher proportion of patients involuntarily [48] and they are a main source of involuntary admissions [49]. GPs working at out-of-hours clinics feel much uncertainty with complex issues and they have little time and few available interventions [26, 47]. Many referring doctors find it difficult to apply the legal criteria [49] and one study [50] found that the danger criterion had been used most often. The most frequent expectations for the involuntary admissions are the starting of treatment with neuroleptics and taking care of the patient [51]. While one study found that referring GPs and admitting specialists were mostly in agreement [45], other studies had found that 27% [52], 45% [46], and 54% [44] were disallowed, i.e. either admitted voluntarily or not admitted at all. One study showed that those who were not received involuntarily had shorter stays [44]. Factors that have been found to predict disallowance were: less symptoms, neurotic and personality disorders, suicide risk, intoxication, and low referring agent competence [52]. One study [27] found that admitting specialists expressed a paternalistic attitude.

### Studies examining population-based rates of involuntary admission and factors that influence such rates

Eight studies examined rates of involuntary admission [14, 53–59] and factors that can influence the rates. In this category, all the included studies had a quantitative design, as expected.

### Main findings

Data were collected from the Norwegian Patient Register [57, 58], from hospital records [54, 55, 59], and from national statistics [53, 56]. Rates varied from 135/100,000 [56] to 418/100,000 [57], depending on sample type and study period. Patients suffering from schizophrenia/psychoses had higher rates of involuntary admission [53, 55]. Males [53, 55] had higher rates than females. Older patients, patients not in paid work, and those living alone had higher rates [53]. People who were living in traditional Sami areas but were not Sami had higher rates than the Sami [57]. One intervention study [14] suggested that by implementing a check list for involuntary admission, and by giving personal feedback to referring doctors, the percentage of involuntary admissions fell from 79% in 2000 to 40% in 2008.

### Studies examining characteristics of involuntarily admitted patients and predictors of involuntary status

Seventeen studies [60–76] concerned the topic of characteristics of involuntarily admitted patients and predictors of involuntary status among admitted patients. All the included articles in this category had a quantitative design.

### Main findings

Being involuntarily admitted was associated with a range of factors, including the diagnosis of schizophrenia [61, 69], psychosis [60, 72], and more severe symptoms [71, 74]. Some studies found that males [60, 71, 74] and patients with less education [69, 71, 74] were more often involuntarily admitted. Being admitted to acute wards [61] or being an emergency admission [72] were also associated with involuntary status. Involuntary admission was also associated with high narcissism scores [64], violence [65], and admission with police assistance [69, 74]. Committed patients were less often of Norwegian origin [62, 74] and refugees were more often involuntary than asylum seekers were [63]. One study found an association with younger age [70], while another found an association with older age [74]. Type of service system also significantly predicted admission [60]. Involuntary patients were less often known by the referring agent [74]. One study found that 35% of the involuntary patients had been secluded and that 10% had been physically restrained [67]. Another study found that restrained patients were more likely to be involuntarily referred [68], however one study found that the number of restraint episodes for those who had been restrained was not associated with legal status [75]. One study [76] showed that 19% of the committed patients were involuntarily medicated. Another study found that women who had always been admitted voluntarily had a higher standardized mortality rate [70]. A study comparing characteristics of patients in one Russian and two Norwegian hospitals found that a higher proportion of patients in the Russian hospital were voluntary [73].

### Studies of staff attitudes to involuntary admission and coercion

Nine articles dealt with the topic of staff attitudes to involuntary admission and coercion [28, 29, 31, 77–82]. Some of the articles dealt specifically with the topic of admission, while others examined more general attitudes, and included admission as one of several types of coercion that were discussed. Six of the studies had different types of questionnaire-based designs [77–82], including questionnaires with fictitious cases [77, 78, 81] and rating scales [79, 82]. Some used individual interviews [28, 29], focus group interviews [31], and/or observation [29]. Six of the included articles in this category had a quantitative design [77–82], while three [28, 29, 31] had a qualitative design.

### Main findings

The mean rate of willingness to coerce varied from 30 to 81.1% [77, 78]. Staff were more willing to coerce when patients were violent [77, 78, 81]. Some staff saw forced medication as a matter of necessity in certain cases [29]. One article identified three main attitudes to coercion; coercion as offending, coercion as security, and coercion as treatment [79]. Most of the variation in attitudes seemed to be attributed to variation between staff within wards [82]. Most staff agreed with a ‘coercion as care and security attitude’ [82]. Roles in healthcare were important for attitudes [80]. Some staff were uncomfortable with using coercion [29, 31].

### Studies examining outcomes of involuntary admission or outcomes of studies aiming to influence involuntary admission

Eight of the included articles [15–17, 83–87] dealt with outcomes of stays involving patients who had been involuntarily admitted specifically or outcomes where the involuntary patients were treated as part of a larger group of patients. All the articles in this category had a quantitative design.

### Main findings

In a study of outcomes of patients with recent-onset schizophrenia [83], patients who adhered to medication had fewer coerced inpatient days. One study of patients at an emergency unit found that while an improved information procedure increased patients’ satisfaction with the information, overall satisfaction was not increased [15]. There were no differences in general satisfaction scores between voluntary and involuntary patients, but the voluntary felt they had been received better (p < 0.002) and had better quality information (< 0.001) than the involuntary. Converting two standard beds into beds that were available without referral reduced the number of coerced bed-days (for those whom the beds were available to) with approximately 50% [16]. A study where patients were given the chance of self-referral found a reduction in the number of involuntary days by 61% [17]. One study found that involuntary patients had more symptoms at intake and improved more during a 2-year follow-up period following a first-time admission for first time psychosis [84]. In another study [85], involuntary admission did not significantly influence outcomes in terms of BPRS scores. One study showed that including specific patient groups in ACT-teams reduced the number of involuntary inpatient days [86, 87].

## Discussion

Despite the relatively high number of articles dealing with the topic of involuntary admission in Norway, the variation in topics, samples, designs, methods and outcomes makes aggregating the findings challenging—an issue that has been discussed also with respect to the international literature [88–90].

The largest category of articles was on Patients’ experiences, including the topics of patients’ satisfaction and perceived coercion. Interestingly, this category also stands out as having the largest proportion of qualitative studies or mixed-method designs (13 of 21 articles). The most common method for data-gathering in this category was interviews (13 of 21 articles), but various qualitative and quantitative approaches were represented. The studies showed that perceived coercion did not equate with involuntary legal status, a finding supported by some of the international literature [1, 91]. However, the EUNOMIA study, which included data from 12 countries, found that involuntary admission was significantly associated with a high level of perceived coercion [7]. While some of the Norwegian studies [38, 39, 41] found that formally coerced patients were less satisfied, the finding was not consistent [42]. Some studies from other countries have also suggested that patients that are formally coerced are less satisfied [90, 92], but also in the international literature this finding has not been consistent [93]. Some of the reviewed Norwegian studies in this category [18, 21, 40, 43] described how patients experienced humiliation and stigma when being committed.

The second largest group of articles (17) concerned Characteristics of involuntary patients and predictors of involuntary status. In several studies, involuntary admission was associated with severe mental illness [60, 61, 69–72, 74], being male [60, 71, 74] and low educational status [69, 71, 74]. Involuntary status was also associated with emergencies [72], patients scoring high on narcissism [64], police involvement [69, 74], non-Norwegian origin of patients [62, 74], patients having refugee status [63], centralized psychiatric beds [60], and patients not being known by the referring doctors [74]. The findings suggest that Norwegian patients that are more severely ill, male, in an emergency situation possibly with some sort of violence and police involvement, are more likely to be committed. Furthermore, being non-Norwegian and being unknown to the referring doctor also seems to increase the likelihood of being committed. These factors could possibly be explained by considering that they either represent difficulties in communication (being unknown and/or non-Norwegian) or that they represent some form of actual or perceived danger (severe illness, emergency situation, male, violence, police involvement), two themes that might be particularly important when GPs make assessment regarding referrals to involuntary admission [26, 51]. Studies from different countries suggest that predictors of involuntary admission may vary between countries, possibly because of differences in cultural factors [94], factors related to the health services, legislation, or other factors. For instance, being male has been associated with an increased risk of involuntary admission in New Zealand, Ireland, and Italy [94–97], but not in China [97]. Also, with respect to other predictors, findings seem to differ much in the international literature [98]. While some studies have suggested that involuntary admission and/or coercion in general is associated with illness severity [95, 98–100], other studies have not reached the same conclusion [90, 101]. The Danish OPUS-trial did not find a significant difference in involuntary admission days between patients in three different treatment models. However, the authors remark that the sample might have been too small to detect actual differences [8]. In sum, the local context appears to be important in understanding predictors of involuntary admission, underlining the need for studies from different countries.

Eleven studies focused on the referral or admission processes specifically. These were mainly studies with a quantitative design, focusing either on the referring doctors or on the hospital-based specialist decisions. The role of the GPs working at out-of-hours clinics seems to be central in the process of referring many of the patients that are committed [48, 49]. The GPs often have little time and few available interventions [26, 47]. Similar stressful working conditions have also been described for GPs in other countries, including Denmark [102]. Studies from other countries have suggested that personal characteristics of GPs can be important to decisions to commit, as can local collaborations with mental health services [103]. The availability of alternatives to hospitalisation, such as acute day care or improved home care nursing, could reduce the need to involuntarily admit patients [6, 104]. While GPs have a very central role in referring involuntary patients in Norway [48, 49], in some other countries, emergency services may be more important as a first point of contact [105]. The proportion of patients that were disallowed by the receiving hospital-based specialists in Norway varied from ‘mostly in agreement’ [45] to approximately half being disallowed [44, 46]. An Australian [106] study found that 40–73% of patients that had been referred (‘certified’) were disallowed. The highest rate (73%) was found for referrals from ambulance officers while the rate for doctors (40%) was more comparable to the Norwegian rates [106].

Staff attitudes to coercion was the topic of nine of the reviewed articles. This category included studies with both a quantitative and qualitative design. The studies suggested that there was much variation in the proportion of staff that was willing to use coercion [77, 78, 82], but that most seemed to agree that coercion might provide both care and security [82]. Staff attitudes to coercion can be important also because they might influence how staff interpret and enforce mental health legislation [107, 108]. The Norwegian studies included in the review as well as studies from other countries have found that a range of factors may impact attitudes to the use of coercion, including age, gender, professional role, work experience, own experiences with mental illness, knowledge, personality, and values [2, 80–82, 109].

Rates of involuntary admission was the topic of eight articles. Rates varied from 135 to 418 per 100,000 [56, 57], depending on which population samples that were studied. The rates were higher with patients suffering from psychotic disorders, patients that were male, older, not working, and living alone [53, 55]. National statistics have suggested average rates of approximately 140/100,000 in recent years [10], but these figures have been criticised for being based on inconsistent reporting [55, 110]. It is not surprising that the rates varied much within Norway, as former studies have suggested that rates of different types of coercion vary within countries and even between comparable hospitals [108]. Such intra-country variation in rates can be explained by differences in study methods, study populations and hospitals/wards and local attitudes to coercion [5, 60, 72, 76, 108, 111–116].

Rates of involuntary hospital admissions vary considerably between countries. Dressing and Salize [117] found that rates varied from 6/100,000 inhabitants in Portugal to 218/100,000 inhabitants in Finland. Compared to these figures, the rates described in the Norwegian studies seem to be on the high end. Differences in rates between countries may in part be due to differences in legislation [118, 119]. Recently, changes have been introduced to the Norwegian legislation, including a requirement of a lack of capacity to consent and for an exhaustive written justification for involuntary admission [12]. These changes could help reduce rates, although such an effect on coercion has not been consistent in other countries that have changed their legislation [120–122].

The category outcomes of involuntary admission comprised eight articles. One study found that first-time psychosis coerced patients improved more than non-coerced patients [84]. This Norwegian study supports the finding from some studies from other countries that involuntary patients’ symptoms improve on the short term and over time [123–125]. Other studies from other countries have, however, found that formal coercion has little or no impact on clinical outcomes [126, 127], but may negatively impact satisfaction and quality of life [90]. Different methodological approaches might explain some of the differences in findings regarding outcomes of involuntary admission [124]. An interesting finding in the Norwegian literature is that self-referral to hospital appears to reduce the rates of coercion [16, 17] as does including patients in ACT-teams [86, 87]. These, in Norway, relatively new services therefore seem to show promise in this respect and should be studied further [128].

A main finding was that, with the exception of four articles, all the identified studies relating to involuntary admission in Norwegian adult psychiatric hospitals were observational. It is clearly more challenging to perform intervention studies in this field of research, especially as ethical concerns and the often acute nature of involuntary admissions pose constraints on research designs [3]. However, the four intervention studies show that it is possible to use such designs, although they might be criticised for flaws (i.e. lack of control groups, etc.). One of the studies [14] implemented changes in procedures among the referring doctors, one study [15] improved patient information routines, and in two studies [16, 17] patients were given more powers in deciding when they should be admitted. As these studies have shown promising results, more high-quality intervention studies would help forwarding this field of research in Norway.

### Strengths and limitations

It is a limitation that the search process was carried out only by one researcher. The search strategy supplemented with the hand searches and searches on some Norwegian websites most likely identified the relevant scientific peer-reviewed studies. However, one cannot rule out that some studies can have been missed because they were not published in journals that were indexed by the databases that were searched and that they were missed in the supplemental searches. As this review is based on the Norwegian literature only, the findings cannot be generalized to other countries.

## Conclusions

Seventy-four studies were included in the review, and a wide variation in topics and approaches were identified, which makes aggregating the findings challenging—an issue that has been discussed also with respect to the international literature. Main topics that were identified were on patients’ experiences, the referral process, rates and characteristics of involuntary patients, staff attitudes, and outcomes. Some studies showed that patients could feel humiliated when involuntarily admitted, but that patients’ legal status did not equate with level of perceived coercion. The severely ill, male and those in an emergency were more likely to be involuntarily admitted. GPs working at out-of-hours clinics were often involved in the admission process, and they typically had little time and few available alternatives. While staff differed in their attitudes to coercion, many saw coercion as an expression of care and security. Admission rates varied between 135 and 418 per 100,000. Overall, the findings were in line with the international literature, although the review supports the notion that Norway has a relatively high rate of involuntary admissions compared to international figures. Four interventional studies were identified. These were of particular interest, as they explored interventions for reducing rates of involuntary admission, such as modifying referring routines, improving patient information procedures, and increasing patients’ say in the admission process. However, these interventional studies also had methodological limitations, and more high-quality interventional studies are needed to forward this field of research in Norway. Additional interventions that might reduce rates and could be explored in future studies are educating staff on patients’ perceptions and providing referring GPs with alternatives to involuntary admission.

## Additional files

**Additional file 1.** Example of search string.

**Additional file 2.** Articles included in the categories patients’ experiences, the referral and admission process, and rates of admission (n = 40).

**Additional file 3.** Articles included in the categories characteristics of patients, staff attitudes, and outcomes (N = 34).

## References

[1] Monahan J, Hoge S, Lidz C, Roth L, Bennett N, Gardner W, Mulvey E (1995). Coercion and commitment: understanding involuntary mental hospital admission. Int J Law Psychiatry.

[2] Steinert T (2007). Ethische Einstellungen zu Zwangsunterbringung und -behandlung schizophrener Patienten [Ethical attitudes towards involuntary admission and involuntary treatment of patients with schizophrenia]. (In German). Psychiatr Prax.

[3] Wynn R (2006). Coercion in psychiatric care: clinical, legal and ethical controversies. Int J Psychiatry Clin Pract..

[4] Wynn R (2015). The use of physical restraint in Norwegian adult psychiatric hospitals. Psychiatry J..

[5] Fiorillo A, De Rosa C, Del Vecchio V, Jurjanz L, Schnall K, Onchev G, Alexiev S, Raboch J, Kalisova L, Mastrogianni A, Georgiadou E, Solomon Z, Dembinskas A, Raskauskas V, Nawka P, Nawka A, Kiejna A, Hadrys T, Torres-Gonzales F, Mayoral F, Björkdahl A, Kjellin L, Priebe S, Maj M, Kallert T (2011). How to improve clinical practice on involuntary hospital admissions of psychiatric patients: suggestions from the EUNOMIA study. Eur Psychiatry..

[6] Kallert TW (2011). Involuntary psychiatric hospitalization: current status and future prospects. Srp Arh Celok Lek.

[7] Fiorillo A, Giacco D, de Rosa C, Kallert T, Katsakou C, Onchev G, Raboch J, Mastrogianni A, del Vecchio V, Luciano M, Gatapano F, Dembinskas A, Nawka P, Kiejna A, Torres-Gonzales F, Kjellin L, Maj M, Priebe S (2012). Patient characteristics and symptoms associated with perceived coercion during hospital treatment. Acta Psychiatr Scand.

[8] Øhlenschlæger J, Thorup A, Petersen L, Jepsen P, Køster A, Munkner R, Nordentoft M (2007). Intensive treatment models and coercion. Nord J Psychiatry.

[9] Norwegian Directorate of Health (2014). Bruk av tvang i psykisk helsevern for voksne i 2013 [The use of coercion in the adult psychiatric services in 2013]. (In Norwegian).

[10] Norwegian Directorate of Health (2016). Bruk av tvang i psykisk helsevern for voksne i 2014 [The use of coercion in the adult psychiatric services in 2014]. (In Norwegian).

[11] The Norwegian Minestry of Health and Care Services. Bedre kvalitet—økt frivillighet. Nasjonal strategi for økt frivillighet i psykiske helsetjenester (2012–2015) [Better quality—more voluntary. National strategy for increasing the voluntary use of mental health services]. (In Norwegian). Oslo, Norway; 2012.

[12] Psykisk helsevernloven [The Mental Health Care Act]. (In Norwegian). https://lovdata.no/dokument/NL/lov/1999-07-02-62.

[13] Liberati A, Altman DG, Tetzlaff J, Mulrow C, Gøtzsche PC, Ioannidis JP, Clarke M, Devereaux PJ, Kleijnen J, Moher D (2009). The PRISMA statement for reporting systematic reviews and meta-analyses of studies that evaluate health care interventions: explanation and elaboration. PLoS Med..

[14] Ness E, Steen O, Reichelt JG, Walby FA (2016). Reduksjon av tvangsinnleggelser fra legevakt [Reduced number of involuntary commitments from an emergency outpatient clinic] (in Norwegian). Tidsskrift for Norsk psykologforening..

[15] Johnsen L, Oysaed H, Børnes K, Moe TJ, Haavik J (2007). A systematic intervention to improve patient information routines and satisfaction in a psychiatric emergency unit. Nord J Psychiatry.

[16] Heskestad S, Tytlandsvik M (2008). Brukerstyrte kriseinnleggelser ved alvorlig psykisk lidelse [Patient-guided crisis admissions for severe psychotic conditions]. (In Norwegian). Tidsskr Nor Laegeforen.

[17] Støvind H, Hanneborg EM, Ruud T (2012). Bedre tid med brukerstyrte innleggelser [More time with patient controlled admissions] (in Norwegian). Sykepleien.

[18] Svindseth MF, Dahl AA, Hatling T (2007). Patients’ experiences of humiliation in the admission process to acute psychiatric wards. Nord J Psychiatry.

[19] Larsen IB, Terkelsen TB (2013). Coercion in a locked psychiatric ward: Perspectives of patients and staff. Nurs Ethics..

[20] Terkelsen TB, Larsen IB (2013). The locked psychiatric ward: hotel or detention camp for people with dual diagnosis. J Mental Health..

[21] Pedersen KL (2008). Pårørendes opplevelse av politiassistert tvangsinnleggelse i psykiatrisk institusjon [Caregivers’ experiences of forensic commitment in mental health] (In Norwegian). Sykepleien Forskning..

[22] Thorvik A (2012). ‘Noen ganger er det kanskje nødvendig’ Om egenopplevelse av tvunget vern ved suicidalitet [‘Some times it might be necessary’. Patients’ experiences of involuntary admission in relation to suicidality]. (In Norwegian). Suicidologi..

[23] Sebergsen K, Norberg A, Talseth AG (2014). Being in a process of transition to psychosis, as narrated by adults with psychotic illnesses acutely admitted to hospital. J Psychiatr Ment Health Nurs.

[24] Lorem GF, Steffensen M, Frafjord J, Wange CEA (2014). Omsorg under tvang. En narrativ studie av pasienters fortellinger om tvang og psykisk helsevern [Compulsory care: a narrative study of patient stories of coercion and mental health care]. (In Norwegian). Tidsskrift for Psykisk Helsearbeid..

[25] Terkelsen TB, Larsen IB (2016). Fear, danger and aggression in a Norwegian locked psychiatric ward: dialogue and ethics of care as contributions to combating difficult situations. Nurs Ethics.

[26] Johansen IH, Carlsen B, Hunskaar S (2011). Psychiatry out-of-hours: a focus group study of GPs’ experiences in Norwegian casualty clinics. BMC Health Serv Res..

[27] Feiring E, Ugstad KN (2014). Interpretations of legal criteria for involuntary psychiatric admission: a qualitative analysis. BMC Health Serv Res..

[28] Mattson ÅM, Binder P-E (2012). A qualitative exploration of how health care workers in an inpatient setting in Norway experience working with patients who self-injure. Nordic Psychol..

[29] Terkelsen TB, Larsen IB (2012). Tvangsmedisinering som permanent unntakstilstand [Compulsory medication as a state of exception: experiences from fieldwork]. (In Norwegian). Tidsskrift for Psykisk Helsebarbeid..

[30] Lorem GF, Hem MH, Molewijk B (2015). Good coercion: patients’ moral evaluation of coercion in mental health care. Int J Ment Health Nurs..

[31] Valenti E, Banks C, Calcedo-Barba A, Bensimon CM, Hoffmann KM, Pelto-Piri V, Jurin T, Mendoza OM, Mundt AP, Rugkåsa J, Tubini J, Priebe S (2015). Informal coercion in psychiatry: a focus group study of attitudes and experiences of mental health professionals in ten countries. Soc Psychiatry Psychiatr Epidemiol.

[32] Kogstad RE (2009). Protecting mental health clients’ dignity—the importance of legal control. Int J Law Psychiatry.

[33] Kogstad RE, Ekeland TJ, Hummelvoll JK (2011). In defence of a humanistic approach to mental health care: recovery processes investigated with the help of clients’ narratives on turning points and processes of gradual change. J Psychiatr Ment Health Nurs.

[34] Norvoll R, Pedersen R (2016). Exploring the views of people with mental health problems’ on the concept of coercion: towards a broader socio-ethical perspective. Soc Sci Med.

[35] Iversen KI, Høyer G, Sexton H, Grønli OK (2002). Perceived coercion among patients admitted to acute wards in Norway. Nord J Psychiatry.

[36] Sørgaard KW (2004). Patients’ perception of coercion in acute psychiatric wards. An intervention study. Nord J Psychiatry..

[37] Kjellin L, Høyer G, Engberg M, Kaltiala-Heino R, Sigurjónsdóttir M (2006). Differences in perceived coercion at admission to psychiatric hospitals in the Nordic countries. Soc Psychiatry Psychiatr Epidemiol.

[38] Sørgaard KW (2007). Satisfaction and coercion among voluntary, persuaded/pressured and committed patients in acute psychiatric treatment. Scand J Caring Sci.

[39] Iversen KI, Høyer G, Sexton HC (2007). Coercion and patient satisfaction on psychiatric acute wards. Int J Law Psychiatry.

[40] Svindseth MF, Nøttestad JA, Dahl AA (2013). Perceived humiliation during admission to a psychiatric emergency service and its relation to socio-demography and psychopathology. BMC Psychiatry..

[41] Bø B, Ottesen ØH, Gjestad R, Jørgensen HA, Kroken RA, Løberg EM, Johnsen E (2016). Patient satisfaction after acute admission for psychosis. Nord J Psychiatry.

[42] Wynn R, Myklebust LH (2006). Patients’ satisfaction and self-rated improvement following coercive interventions. Psychiatry Psychol Law.

[43] Svindseth MF (2015). Opplevd krenkelse i innleggelsessituasjonen til akuttpsykiatrisk avdeling og assosiasjoner til negative hendelser samt kjønnsforskjeller [Perceived humiliation in the admission process to psychiatric care and associations to negative situations and gender differences]. (In Norwegian). Nordic J Nursing Res..

[44] Gjelstad K, Løvdal H, Ruud T, Friis S (2003). Tvangsinnleggelser til psykiatrisk observasjon—blir de opphevet dagen etter? [Compulsory admissions for observation in emergency psychiatric departments–discharge next day?]. (In Norwegian). Tidsskr Nor Laegeforen.

[45] Deraas TS, Hansen V, Giaever A, Olstad R (2006). Acute psychiatric admissions from an out-of-hours casualty clinic; how do referring doctors and admitting specialists agree?. BMC Health Serv Res..

[46] Tørrissen T (2007). Tvangsinnleggelser i en akuttpsykiatrisk post. Involuntary admissions to an acute psychiatric ward [Article in Norwegian]. Tidsskr Nor Laegeforen.

[47] Johansen IH, Morken T, Hunskaar S (2012). How Norwegian casualty clinics handle contacts related to mental illness: a prospective observational study. Int J Ment Health Syst..

[48] Johansen IH, Mellesdal L, Jørgensen HA, Hunskaar S (2012). Admissions to a Norwegian emergency psychiatric ward: patient characteristics and referring agents. A prospective study. Nord J Psychiatry..

[49] Røtvold K, Wynn R (2015). Involuntary psychiatric admission: characteristics of the referring doctors and the doctors’ experiences of being pressured. Nord J Psychiatry.

[50] Røtvold K, Wynn R (2015). Involuntary psychiatric admission: the referring general practitioners’ assessment of patients’ dangerousness and need for psychiatric hospital treatment. Nord J Psychiatry.

[51] Røtvold K, Wynn R (2016). Involuntary psychiatric admission: how the patients are detected and the general practitioners’ expectations for hospitalization. An interview-based study. Int J Ment Health Syst..

[52] Fuglseth NL, Gjestad R, Mellesdal L, Hunskaar S, Oedegaard KJ, Johansen IH (2016). Factors associated with disallowance of compulsory mental healthcare referrals. Acta Psychiatr Scand.

[53] Hatling T, Krogen T, Ulleberg P (2002). Compulsory admissions to psychiatric hospitals in Norway—international comparisons and regional variations. J Ment Health..

[54] Berg JE, Johnsen E (2004). Innlegges innvandrere oftere enn etniske nordmenn i akuttpsykiatriske avdelinger? [Are admission rates to acute psychiatric care higher for immigrants from non-Western countries than for the traditional Norwegian population?]. (In Norwegian). Tidsskr Nor Lægeforen..

[55] Iversen KI, Høyer G, Sexton HC (2009). Rates for civil commitment to psychiatric hospitals in Norway. Are registry data accurate?. Nord J Psychiatry.

[56] Bak J, Aggernæs H (2012). Coercion within Danish psychiatry compared with 10 other European countries. Nord J Psychiatry.

[57] Norum J, Bjerke FE, Nybrodahl I, Olsen A (2012). Admission and stay in psychiatric hospitals in northern Norway among Sami and a control group. Nord J Psychiatry.

[58] Norum J, Olsen A, Nybrodahl I, Sørgaard KW (2013). Compulsory and voluntary admission in psychiatric hospitals in northern Norway 2009–2010. A national registry-based analysis. Nord J Psychiatry.

[59] Tøgersen K, Bjerke E, Gjelstad K, Ruud T (2015). Psykiatriske tvangsinnleggelser i Østfold i 2000 og 2010 [Compulsory hospitalisation in mental health care in Østfold in 2000 and 2010]. (In Norwegian). Tidsskr Nor Laegeforen.

[60] Myklebust LH, Sørgaard K, Wynn R (2014). Local psychiatric beds appear to decrease the use of involuntary admission: a case-registry study. BMC Health Serv Res..

[61] Bjørngaard JH, Heggestad T (2001). Kan ulik pasientsammensetning forklare forskjeller i tvangsinnleggelser? [Can case-mix explain differences in involuntary admissions to Norwegian psychiatric hospitals?] (In Norwegian). Tidsskr Nor Laegeforen.

[62] Iversen VC, Morken G (2003). Acute admissions among immigrants and asylum seekers to a psychiatric hospital in Norway. Soc Psychiatry Psychiatr Epidemiol.

[63] Iversen VC, Morken G (2004). Differences in acute psychiatric admissions between asylum seekers and refugees. Nord J Psychiatry.

[64] Svindseth MF, Nøttestad JA, Wallin J, Roaldset JO, Dahl AA (2008). Narcissism in patients admitted to psychiatric acute wards: its relation to violence, suicidality and other psychopathology. BMC Psychiatry..

[65] Roaldset JO, Bakken AM, Bjørkly S (2011). A prospective study of lipids and serotonin as risk markers of violence and self-harm in acute psychiatric patients. Psychiatry Res.

[66] Roaldset JO, Bjørkly S (2010). Patients’ own statements of their future risk for violent and self-harm behaviour: a prospective inpatient and post-discharge follow-up study in an acute psychiatric unit. Psychiatry Res.

[67] Husum TL, Bjørngaard JH, Finset A, Ruud T (2010). A cross-sectional prospective study of seclusion, restraint and involuntary medication in acute psychiatric wards: patient, staff and ward characteristics. BMC Health Serv Res..

[68] Knutzen M, Mjosund NH, Eidhammer G, Lorentzen S, Opjordsmoen S, Sandvik L, Friis S (2011). Characteristics of psychiatric inpatients who experienced restraint and those who did not: a case–control study. Psychiatr Serv.

[69] Opsal A, Kristensen Ø, Ruud T, Larsen TK, Gråwe RW, Clausen T (2011). Substance abuse in patients admitted voluntarily and involuntarily to acute psychiatric wards: a national cross-sectional study. Norsk Epidemiol..

[70] Høye A, Jacobsen BK, Hansen V (2011). Increasing mortality in schizophrenia: are women at particular risk? A follow-up of 1111 patients admitted during 1980–2006 in Northern Norway. Schizophr Res.

[71] Iversen VC, Berg JE, Småvik R, Vaaler AE (2011). Clinical differences between immigrants voluntarily and involuntarily admitted to acute psychiatric units: a 3-year prospective study. J Psychiatr Ment Health Nurs.

[72] Myklebust LH, Sørgaard K, Røtvold K, Wynn R (2012). Factors of importance to involuntary admission. Nord J Psychiatry.

[73] Sørgaard KW, Rezvy G, Bugdanov A, Sørlie T, Bratlid T (2013). Treatment needs, diagnoses and use of services for acutely admitted psychiatric patients in northwest Russia and northern Norway. Int J Ment Health Syst..

[74] Hustoft K, Larsen TK, Auestad B, Joa I, Johannessen JO, Ruud T (2013). Predictors of involuntary hospitalizations to acute psychiatry. Int J Law Psychiatry.

[75] Knutzen M, Bjørkly S, Eidhammer G, Lorentzen S, Mjøsund NH, Opjordsmoen S, Sandvik L, Friis S (2014). Characteristics of patients frequently subjected to pharmacological and mechanical restraint—a register study in three Norwegian acute psychiatric wards. Psychiatry Res.

[76] Christensen TB, Onstad S (2003). Tvangsbehandling med legemidler i en psykiatrisk akuttavdeling [Compulsory medical treatment in an emergency psychiatric department]. (In Norwegian). Tidsskr Nor Laegeforen.

[77] Wynn R, Myklebust LH, Bratlid T (2006). Attitudes to coercion among health-care workers and the general public in Norway. J Psychiatr Intensive Care.

[78] Wynn R, Myklebust LH, Bratlid T (2007). Psychologists and coercion: decisions regarding involuntary psychiatric admission and treatment in a group of Norwegian psychologists. Nord J Psychiatry.

[79] Husum TL, Finset A, Ruud T (2008). The staff attitude to coercion scale (SACS): reliability, validity and feasibility. Int J Law Psychiatry.

[80] Diseth RR, Bøgwald KP, Høglend PA (2011). Attitudes among stakeholders towards compulsory mental health care in Norway. Int J Law Psychiatry.

[81] Wynn R, Kvalvik AM, Hynnekleiv T (2011). Attitudes to coercion at two Norwegian psychiatric units. Nord J Psychiatry.

[82] Husum TL, Bjørngaard JH, Finset A, Ruud T (2011). Staff attitudes and thoughts about the use of coercion in acute psychiatric wards. Soc Psychiatry Psychiatr Epidemiol.

[83] Morken G, Widen JH, Grawe RW (2008). Non-adherence to antipsychotic medication, relapse and rehospitalisation in recent-onset schizophrenia. BMC Psychiatry..

[84] Opjordsmoen S, Friis S, Melle I, Haahr U, Johannessen JO, Larsen TK, Røssberg JI, Rund BR, Simonsen E, Vaglum P, McGlashan TH (2010). A 2-year follow-up of involuntary admission’s influence upon adherence and outcome in first-episode psychosis. Acta Psychiatr Scand.

[85] Svindseth MF, Nøttestad JA, Dahl AA (2010). A study of outcome in patients treated at a psychiatric emergency unit. Nord J Psychiatry.

[86] Clausen H, Ruud T, Odden S, Šaltytė Benth J, Heiervang KS, Stuen HK, Killaspy H, Drake RE, Landheim A (2016). Hospitalisation of severely mentally ill patients with and without problematic substance use before and during assertive community treatment: an observational cohort study. BMC Psychiatry..

[87] Clausen H, Landheim A, Odden S, Šaltytė Benth J, Heiervang KS, Stuen HK, Killaspy H, Ruud T (2016). Hospitalization of high and low inpatient service users before and after enrollment into assertive community treatment teams: a naturalistic observational study. Int J Ment Health Syst..

[88] Janssen WA, van de Sande R, Noorthoorn EO, Nijman HL, Bowers L, Mulder CL, Smit A, Widdershoven GA, Steinert T (2011). Methodological issues in monitoring the use of coercive measures. Int J Law Psychiatry.

[89] Martin V, Kuster W, Baur M, Bohnet U, Hermelink G, Knopp M, Kronstorfer R, Martinez-Funk B, Roser M, Voigtländer W, Brandecker R, Steinert T (2007). Die Inzidenz von Zwangsmaßnahmen als Qualitätsindikator in psychiatrischen Kliniken. Probleme der Datenerfassung und -verarbeitung und erste Ergebnisse [Incidence of coercive measures as an indicator of quality in psychiatric hospitals. Problems of data recording and processing, preliminary results of a benchmarking study]. (In German). Psychiatr Prax.

[90] Kallert TW, Glöckner M, Schützwohl M (2008). Involuntary vs. voluntary hospital admission. A systematic literature review on outcome diversity. Eur Arch Psychiatry Clin Neurosci.

[91] Katsakou C, Maroucka S, Garabette J, Rost F, Yeeles K, Priebe S (2011). Why do some voluntary patients feel coerced into hospitalization?. A mixed-methods study. Psychiatry Res..

[92] Strauss JL, Zervakis JB, Stechuchak KM, Olsen MK, Swanson J, Swartz MS, Weinberger M, Marx CE, Calhoun PS, Bradford DW, Butterfield MI, Oddone EZ (2013). Adverse impact of coercive treatments on psychiatric inpatients’ satisfaction with care. Community Ment Health J.

[93] O’Donoghue B, Roche E, Shannon S, Creed L, Lyne J, Madigan K, Feeney L (2015). Longer term outcomes of voluntarily admitted service users with high levels of perceived coercion. Psychiatry Res.

[94] Curley A, Agada E, Emechebe A, Anamdi C, Ng XT, Duffy R, Kelly BD (2016). Exploring and explaining involuntary care: the relationship between psychiatric admission status, gender and other demographic and clinical variables. Int J Law Psychiatry.

[95] Wheeler A, Robinson E, Robinson G (2005). Admissions to acute psychiatric inpatient services in Auckland, New Zealand: a demographic and diagnostic review. NZ Med J.

[96] Donisi V, Tedeschi F, Salazzari D, Amaddeo F (2016). Differences in the use of involuntary admission across the Veneto Region: which role for individual and contextual variables?. Epidemiol Psychiatr Sci..

[97] Gou L, Zhou JS, Xiang YT, Zhu XM, Correll CU, Ungvari GS, Chiu HF, Lai KY, Wang XP (2014). Frequency of involuntary admissions and its associations with demographic and clinical characteristics in China. Arch Psychiatr Nurs.

[98] Kalisova L, Raboch J, Nawka A, Sampogna G, Cihal L, Kallert TW, Onchev G, Karastergiou A, del Vecchio V, Kiejna A, Adamowski T, Torres-Gonzales F, Cervilla JA, Priebe S, Giacco D, Kjellin L, Dembinskas A, Fiorillo A (2014). Do patient and ward-related characteristics influence the use of coercive measures?. Soc Psych Psychiatr Epidemiol..

[99] Ritsner M, Kurs R, Grinshpoon A (2014). Short-term hospitalization underlies the similarity between involuntarily and voluntarily admitted patients: a 1-year cohort study. Int J Mental Health..

[100] Myklebust LH, Sørgaard K, Wynn R (2017). How mental health service systems are organized may affect the rate of acute admissions to specialized care: report from a natural experiment involving 5338 admissions. SAGE Open Med..

[101] Roelandt J-L, Crétin A, Askevis-Leherpeux F, Baucheron J-P, Brun-Rousseau H, Coldefy M, Daoud V, Defromont L, Giordana J-Y, Maillard I, Roguet J, Saint-Jean H, Thalassinos M, Triantafyllou M, Varomme S, Béhal H, Baleige A, Duhamel A (2017). Compulsory hospitalization, severity of disorders and territorial landscape: a French study. Global J Health Sci..

[102] Jepsen B, Lomborg K, Engberg M (2010). GPs and involuntary admission: a qualitative study. Br J Gen Pract.

[103] Oud MJT, Schuling J, Sloff CJ, Meyboom-deJong B (2007). How do general practitioners experience providing care for their psychotic patients?. BMC Fam Pract..

[104] Røtvold K, Wynn R (2017). How involuntary admission might have been avoided: an interview study of referring general practitioners. Eur Psychiatry..

[105] Anderson KK, Fuhrer R, Malla AK (2010). The pathways to mental health care in first-episode psychosis patients: a systematic review. Psychol Med.

[106] Cutler D, Smith M, Wand T, Green T, Michael D, Gribble R (2013). Involuntary admissions under the Mental Health Act 2007 (New South Wales): a comparison of patients detained by ambulance officers, medical practitioners and accredited persons in an emergency department. Emerg Med Australas..

[107] Wynn R (2003). Staff’s attitudes to the use of restraint and seclusion in a Norwegian university psychiatric hospital. Nord J Psychiatry.

[108] Wynn R (2004). Restraint and seclusion in a Norwegian university psychiatric hospital.

[109] Jaeger M, Ketteler D, Rabenschlag F, Theodoridou A (2014). Informal coercion in acute inpatient setting—knowledge and attitudes held by mental health professionals. Psychiatry Res.

[110] Brofoss KE, Larsen F. (eds.). Evaluering av opptrappingsplanen for psykisk helse (2001-2009). Sluttrapport—syntese og analyse av evalueringens delprosjekter [The evaluation of the escalation plan for mental health (2001–2009). Final report—synthesis and analysis of the sub projects of the evaluation]. (In Norwegian). Oslo: Norwegian Research Council. 2009. http://www.forskningsradet.no/servlet/Satellite?blobcol=urldata&blobheader=application%2Fpdf&blobheadername1=Content-Disposition%3A&blobheadervalue1=+attachment%3B+filename%3DRAPPORTEVALPSYKISKHELSE2.pdf&blobkey=id&blobtable=MungoBlobs&blobwhere=1274505118054&ssbinary=true.

[111] Lay B, Nordt C, Rössler W (2011). Variation in use of coercive measures in psychiatric hospitals. Eur Psychiatry..

[112] Lepping P, Steinert T, Gebhardt RP, Rüdiger Röttgers H (2004). Attitudes of mental health professionals and lay-people towards involuntary admission and treatment in England and Germany—a questionnaire analysis. Eur Psychiatry.

[113] Luchins DJ, Cooper AE, Hanrahan P, Rasinski K (2004). Psychiatrists’ attitudes toward involuntary hospitalization. Psychiatr Serv.

[114] Swartz MS, Swanson JW, Wagner HR, Hannon MJ, Burns BJ, Shumway M (2003). Assessment of four stakeholder groups’ preferences concerning outpatient commitment for persons with schizophrenia. Am J Psychiatry.

[115] Mulder CL, Uitenbroek D, Broer J, Lendemeijer B, van Veldhuizen JR, van Tilburg W, Lelliott P, Wierdsma AI (2008). Changing patterns in emergency involuntary admissions in the Netherlands in the period 2000–2004. Int J Law Psychiatry.

[116] Hjemås G. Tvang i psykisk helsevern. Geografiske forskjeller i tvangsinnleggelser [Coercion in mental health services. Geographical differences in involuntary admissions]. (In Norwegian). Samfunnsspeilet. 2011. https://www.ssb.no/helse/artikler-og-publikasjoner/geografiske-forskjeller-i-tvangsinnleggelser.

[117] Dressing H, Salize HJ (2004). Compulsory admission of mentally ill patients in European Union Member States. Soc Psychiatr Psychiatr Epidemiol..

[118] Kieber-Ospelt I, Theodoridou A, Hoff P, Kawohl W, Seifritz E, Jaeger M (2016). Quality criteria of involuntary psychiatric admissions—before and after the revision of the civil code in Switzerland. BMC Psychiatry..

[119] Zhang S, Mellsop G, Brink J, Wang X (2015). Involuntary admission and treatment of patients with mental disorder. Neurosci Bull..

[120] Nijman H, Campo JMLG, Ravelli DP (1999). Increase in the number of involuntary admissions. Tijdschrift Voor Psychiatrie..

[121] Keski-Valkama A, Eronen M, Koivisto A-M, Lönnqvist J, Kaltiala-Heino R (2007). A 15-year national follow-up: legislation is not enough to reduce the use of seclusion and restraint. Soc Psychiatry Psychiatr Epidemiol.

[122] Bagby RM, Atkinson L (1988). The effects of legislation reform on civil commitment rates: a critical analysis. Behav Sci Law.

[123] Kallert TW, Katsakou C, Adamowski T, Dembinskas A, Fiorillo A, Kjellin L, Mastrogianni A, Nawka P, Onchev G, Raboch J, Schützwohl M, Solomon Z, Torres-González F, Bremner S, Priebe S (2011). Coerced hospital admission and symptom change—a prospective observational multi-centre study. PLoS ONE.

[124] Katsakou C, Priebe S (2006). Outcomes of involuntary hospital admission—a review. Acta Psych Scand..

[125] Priebe S, Katsakou C, Yeeles K, Amos T, Morriss R, Wang D, Wykes T (2011). Predictors of clinical and social outcomes following involuntary hospital admission: a prospective observational study. Eur Arch Psychiatry Clin Neurosci.

[126] Wallsten T, Kjellin L, Lindström L (2006). Short-term outcome of inpatient psychiatric care—impact of coercion and treatment characteristics. Soc Psychiatr Psychiatr Epidemiol..

[127] Luciano M, Sampogna G, Del Vecchio V, Pingani L, Palumbo C, De Rosa C, Catapano F, Fiorillo A (2014). Use of coercive measures in mental health practice and its impact on outcome: a critical review. Expert Rev Neurother.

[128] Stuen HK, Rugkåsa J, Landheim A, Wynn R (2015). Increased influence and collaboration: a qualitative study of patients’ experiences of community treatment orders within an assertive community treatment setting. BMC Health Serv Res..

