# Supplementary material for: Involuntary admission in Norwegian adult psychiatric hospitals: a systematic review
Source: Int J Ment Health Syst. 2018 Mar 22;12:10. doi: 10.1186/s13033-018-0189-z (PMC5865388; doi:10.1186/s13033-018-0189-z)
Supplement: Supplementary file 1 — Additional file 1. Example of search string. [file 13033_2018_189_MOESM1_ESM.docx]

**Additional file 1**

Search string used for PubMed:

(((refer* or admit or admission or hospitali* or inpatient) and (involuntary or non-voluntary or compuls* or forced or coercion or coerced or detain* or detention) and (Norway or Norwegian or Nordic or Scandinav*)) not (child* or

adolescent) not (rat or animal) not (lung or pulmonary or asthma))
