# Supplementary material for: Involuntary admission in Norwegian adult psychiatric hospitals: a systematic review
Source: Int J Ment Health Syst. 2018 Mar 22;12:10. doi: 10.1186/s13033-018-0189-z (PMC5865388; doi:10.1186/s13033-018-0189-z)
Supplement: Supplementary file 2 — Additional file 2. Articles included in the categories patients’ experiences, the referral and admission process, and rates of admission (n = 40). [file 13033_2018_189_MOESM2_ESM.docx]

**Additional file 2.**

**Table 1. Articles included in the categories patients’ experiences, the referral and admission process, and rates of admissio (n=40)**

| **Category** | **Articles (Authors)** | **Methods/participants (n)** | **Key findings and measures** |
| --- | --- | --- | --- |
| Patients’ experiences | Perceived coercion among patients admitted to acute wards in Norway (Iversen et al., 2002). | Observational, prospective. Interviews and Coercion Ladder/MacArthur Admission Experience Scale of 223 patients. | Involuntary patients (n=150) had significantly higher mean perceived scores. |
| Patients’ experiences | Patients’ perception of coercion in acute psychiatric wards (Sørgaard, 2004). | Observational, prospective. 190 patients completed questionnaires including the Coercion Ladder. | The average value of experienced coercion was low. The main predictor of perceived coercion was seclusion. |
| Patients’ experiences | Differences in perceived coercion at admission to psychiatric hospitals in the Nordic countries (Kjellin et al., 2006). | Observational, prospective. 426 involuntary and 494 voluntary patients in 12 psychiatric hospitals in the Nordic countries were interviewed and assessed. | The proportion of involuntarily admitted patients that reported perceived coercion varied from 49% in Norway to 100% in Iceland. |
| Patients’ experiences | Patients’ experiences of humiliation in the admission process to acute psychiatric wards (Svindseth et al., 2007). | Observational, prospective. 102 patients admitted to acute wards were interviewed. Six patients were qualitatively interviewed. | Patients’ conviction that ‘the admission was not right’ significantly predicted feeling of humiliation (B=-2.91, Beta=-0.33, p=0.004). |
| Patients’ experiences | Satisfaction and coercion among voluntary, persuaded/pressured and committed patients in acute psychiatric treatment (Sørgaard, 2007). | Observational, prospective.189 patients in two acute wards answered questionnaires. | Committed patients were less satisfied with the treatment than the persuaded or the voluntary (3.69 vs. 4.02 and 4.26, p=0.02). |
| Patients’ experiences | Coercion and patient satisfaction on psychiatric acute wards (Iversen et al., 2007) | Observational, prospective.173 patients in three acute wards were administered the Nordic Admission Interview. 94 completed a satisfaction questionnaire. | 45% had high levels of perceived coercion, no significance was found for legal status. Overall satisfaction was negatively impacted by accumulated coercive events. |
| Patients’ experiences | Protecting mental health clients’ dignity (Kogstad, 2009) | Qualitative content analysis of 267 client narratives regarding the violation of dignity. | Negative experiences were grouped into main categories: miscommunication (58), rejection (104) and humiliation/punishment (105). |
| Patients’ experiences | In defence of a humanistic approach to mental health care (Kogstad et al., 2011) | Observational, retrospective. Qualitative content analysis of 347 client narratives concerning positive meetings with the health service that constituted a turning point. | Recovery is a personal process that involves finding a new sense of self and feeling of hope, and requires external, material and psychosocial conditions that facilitate the process. |
| Patients’ experiences | Perceived humiliation during admission to a psychiatric emergency service and its relation to socio-demography and psychopathology (Svindseth et al., 2013) | Observational, prospective.186 patients (98 involuntary, 88 voluntary) admitted to two acute wards were interviewed. | 35% were categorized as in the high humiliation group. Compulsory admission, not being in paid work, high scores on BRPS hostility and NPI high entitlement all predicted a high level of humiliation. |
| Patients’ experiences | Coercion in a locked psychiatric ward (Larsen & Terkelsen, 2013) | Observational, prospective.12 patients and 22 staff were included in interviews and/or participant observation in a locked psychiatric ward. | Four analytical themes were identified: 1) Corrections and house rules, 2) Coercion is perceived as necessary, 3) Significance of material surroundings, 4) Being treated as a human being. |
| Patients’ experiences | The locked psychiatric ward (Terkelsen & Larsen, 2013) | Observational, prospective.16 patients with dual diagnoses and 22 staff were included in interviews and/or participant observation in a locked psychiatric ward. | Main themes: 1) the ward as a hotel and 2) the ward as a detention camp. The patients and the staff had different perceptions of involuntary commitment. |
| Patients’ experiences | Patient satisfaction and acute admission for psychosis (Bø et al., 2016). | Observational.104 patients acutely admitted for psychosis completed the UKU Consumer Satisfaction Rating Scale at discharge/follow up. | 88.4% were satisfied. Voluntary patients (mean 9.84, SD=4.82) were more satisfied than involuntary (mean 6.36, SD=8.39) (95%CI=0.84-6.12, p=0.01). |
| Patients’ experiences | Exploring the views of people with mental health problems on the concept of coercion (Norvoll & Pedersen, 2016). | Observational. Three focus group interviews and five individual interviews were conducted with current or prior patients and analysed with thematic content analysis. | Four main themes were identified: Coercion as a wide-ranging phenomenon, coercion as deprivation of freedom, coercion as power relations, coercion as social and existential life events. |
| Patients’ experiences | Caregivers’ experiences of forensic commitment in mental health  (Pedersen, 2008). | Qualitative analysis inspired by Ricoeur of 27 narratives by patients and their caregivers. | The involvement of police in fetching patients at home can represent trauma, debasement, and stigma. |
| Patients’ experiences | ‘Noen ganger er det kanskje nødvendig’ Om egenopplevelse av tvunget vern ved suicidalitet (Thorvik, 2008). | Phenomenological analysis of interviews with 40 inpatients (some of which were involuntarily admitted) suffering from suicidal thoughts. | The informants communicated their retrospective understanding of the necessity for the precaution, i.e. being taken care of in the middle of a suicidal process. |
| Patients’ experiences | Patients’ satisfaction and self-rated improvement following coercive interventions (Wynn et al., 2006) | Questionnaire study of 410 inpatients. Case-control analysis of patients’ assessment of treatment. | No significant difference between coerced (medication, restraint, seclusion) and non-coerced patients. |
| Patients’ experiences | Being in a process of transition to psychosis (Sebergsen et al., 2014). | Narrative interviews with 12 patients with psychosis admitted to acute psychiatric wards. | Patients who did not perceive the signs of psychosis and did not have other people to advocate for them were at risk for coercive interventions. |
| Patients’ experiences | Good coercion: Patients’ moral evaluation of coercion in mental health care (Lorem et al., 2015). | Qualitative inductive study. Participant observation, interviews with 5 patients, focus group discussions with nurses in an acute ward. | Patients’ moral evaluation of coercion. Three themes emerged: agreeing and accepting, fighting or resisting, resignation. |
| Patients’ experiences | Omsorg under tvang (Lorem et al., 2014). | Qualitative interviews with 9 patients that had been committed due to psychosis. Narrative analysis. | The patients expressed understanding for the use of coercion in some instances, but wanted the coercion to be reduced to a minimum. |
| Patients’ experiences | Perceived humiliation in the admission process to psychiatric care and associations to negative situations and gender differences (Svindseth, 2015). | 186 patients being admitted to an acute unit self-rated humiliation on a Cantril ladder. | Involuntary patients significantly more often (Cohens d=0.61, X2=17.77, df=1, p<0.001) than voluntary patients felt humiliated. |
| Patients’ experiences | Fear, danger and aggression in a Norwegian locked psychiatric ward (Terkelsen & Larsen, 2016). | Observational, prospective qualitative study. 12 patients and 22 staff. Interviews and/or participant observation. | The patients and the staff had different perceptions of what might trigger dangerous situations. |
| The referral and admission process | Compulsory admissions for observation in acute psychiatric wards (Gjelstad et al., 2003). | Observational, retrospective.181 patients referred to involuntary observation at an acute psychiatric ward during a three-month period. | 98 patients (54%) were accepted for involuntary observation. Those who were not accepted for involuntary observation had shorter stays. |
| The referral and admission process | Acute psychiatric admission from an out-of-hours Casualty Clinic (Deraas et al., 2006). | Observational. Records of 101 acute psychiatric referrals were examined. | The referring GPs and the hospital specialists mostly agreed on diagnoses and the use of the Mental Health Care Act. |
| The referral and admission process | Involuntary admissions to an acute psychiatric ward (Tørrissen, 2007). | Records of all patients (104) discharged from an acute psychiatric ward during a 6-month period in 2005 were examined. | 49 patients (47%) were referred involuntarily, of which 22 (45%) were admitted voluntarily following specialist assessment. |
| The referral and admission process | Psychiatry out-of-hours (Johansen et al., 2011). | Observational. Qualitative study, thematic approach. 45 GPs that participated in focus groups and individual interviews about challenges in the acute treatment of mental illness and substance abuse in out-of-hours clinics. | The threat to personal safety and security precautions was one main topic. The other main topic was that many of the GPs expressed uncertainty, increased by the complexity of the issues, little time and available information and a limited range of available interventions. |
| The referral and admission process | How Norwegian casualty clinics handle contacts related to mental illness (Johansen et al., 2012). | Observational. Records of 715 nurse-patient contacts and 887 GP-patient contacts related to mental illness or substance abuse at four casualty clinics were examined and compared to other types of contacts at the clinics. | The nurses judged the mental illness/substance abuse contacts to be more urgent and more frequent during night time. When GPs were involved, the most common interventions were lab tests, consulting others regarding treatment, and give medication. |
| The referral and admission process | Admissions to a Norwegian emergency psychiatric ward (Johansen et al., 2012). | In a prospective observational study, records of all admissions to a psychiatric acute clinic during a three-year period were examined (n=5317). | There was no statistically significant difference between referring agents in proportion of involuntarily admitted patients (X^2^=5.77, p<0.12). |
| The referral and admission process | Involuntary psychiatric admission: Characteristics (Røtvold & Wynn, 2015). | Observational. Interview study of 74 GPs that had referred patients to involuntary admission at one major psychiatric hospital. | 45 (61%) had felt pressured to refer the patient, while 34 (49%) had felt pressured to do so involuntarily. 33 (45%) felt that it was difficult to apply the treatment criterion, while 38 (53%) felt it was difficult to apply the danger criterion. |
| The referral and admission process | Involuntary psychiatric admission: The referring (Røtvold & Wynn, 2015). | Observational. Interview study of 74 GPs that had referred patients to involuntary admission at one major psychiatric hospital. | 38% (28) had applied the danger criterion only and 23% (17) had applied the treatment criterion only. 32% (24) had applied both criteria. 74% (55) said that they could not have applied a different criterion. |
| The referral and admission process | Involuntary psychiatric admission: How the patients are detected (Røtvold & Wynn, 2016). | Observational. Interview study of 74 GPs that had referred patients to involuntary admission at one major psychiatric hospital. | 52% (39) were detected by other branches of the health service, 25% (19) by family and 13 (17%) by the police. The expectations for the hospital stay were starting neuroleptics, taking care of the patient, extensive changes to the treatment regime, solve an acute situation, and clarify the diagnosis. |
| The referral and admission process | Factors associated with disallowance of compulsory mental healthcare referrals (Fugleseth et al., 2016). | Observational. Prospective, longitudinal cohort study where referrals, admissions, and patient characteristics were retrieved on compulsory admissions during a three-year period. | 764 of 2813 compulsory admissions were disallowed. Increased GAF symptom score was the strongest predictor of disallowance, as were reported suicide risk, some diagnostic categories, substance intoxication, and low referring agent competence. |
| The referral and admission process | Interpretations of legal criteria for involuntary psychiatric admission (Feiring & Ugstad, 2014). | In-depth interviews with 10 clinicians analysed with deductive thematic method. | A paternalistic perspective was clearly expressed in the data. Involuntary admission was considered to be in the patient’s best interest. |
| Rates of admission | Reduksjon av tvangsinnleggelser fra legevakt (Ness et al., 2016). | Intervention study at a psychiatric out-of-hours clinic with the implementation of a checklist for involuntary admissions and personal feedback to referring doctors.  (Uncontrolled). | The percentage of involuntary referrals fell from 79% in 2000 to 40% in 2008. |
| Rates of admission | Compulsory admission to psychiatric hospitals in Norway (Hatling et al., 2002) | Observational. Data from national statistics on admissions to psychiatric hospitals in Norway in 1996. 4 of 19 counties were excluded due to missing data. | 47% were involuntary and the rate of involuntary admissions was 147/100 000 inhabitants. Patients suffering from psychosis were more likely to be compulsory admitted (61%) than other groups (30%). Younger patients and women were more often voluntarily admitted. |
| Rates of admission | Are admission rates to acute psychiatric care higher for immigrants from non-Western countries than for the traditional Norwegian population? (Berg & Johnsen, 2004) | Observational. Records of patients admitted to one psychiatric ward were examined retrospectively. | Of 415 patients, 80 had immigrant background. Admission rates were 0.0049 for immigrants and 0.0052 for non-immigrants. Immigrants were more often admitted involuntarily. |
| Rates of admission | Rates for civil commitment to psychiatric hospitals in Norway (Iversen et al., 2009). | Observational. Records of admissions to four hospitals during a 6 to 10 month period were examined. 2043 referrals involving 1501 patients were included. | The rate for involuntary admission of patients was 186/100 000 (range 137-255). Males and patients with schizophrenia were more often involuntarily admitted. |
| Rates of admission | Coercion within Danish psychiatry compared with 10 other European countries (Bak & Aggernaes, 2012) | Observational. Review of literature and questionnaire study in 11 European countries. | The rate of forced admissions in Norway in 2001 (135/100 000) was the second highest, following Finland (218/100 000). |
| Rates of admission | Admission and stay in psychiatric hospitals in northern Norway among Sami and a control group (Norum et al., 2012). | Observational. Retrospective study of data from Norwegian Patient Register (2009-2010) of patients admitted from eight predominantly Sami municipalities and 11 control municipalities in north Norway. | Of 1785 admissions, 825 were from the Sami areas. The overall rate of coercion for north Norway was 270/100 000, while it was 384/100 000 in the Sami group and 418/100 000 in the non-Sami group. |
| Rates of admission | Compulsory and voluntary admission in hospitals in Northern Norway 2009-2010 (Norum et al., 2013). | Observational. Retrospective study of data from Norwegian Patient Register (2009-2010) of patients admitted in north Norway. | 8613 admissions were included. The rates of decisions on any form of coercion related to admission were in total 210/100 000 (range 100/100 000-350/100 000). |
| Rates of admission | Compulsory hospitalisation in mental health care in Østfold in 2000 and 2010 (Tøgersen et al., 2015). | Observational. A partially prospective study of emergency admissions to a psychiatric hospital in two periods (3 months in 2000 and in 2010). | There was a significant increase in the number of acute admissions. The rate of involuntary patients was not statistically different in the two periods but the percentage of involuntary admissions fell from 37% to 26%. |
