# Supplementary material for: Involuntary admission in Norwegian adult psychiatric hospitals: a systematic review
Source: Int J Ment Health Syst. 2018 Mar 22;12:10. doi: 10.1186/s13033-018-0189-z (PMC5865388; doi:10.1186/s13033-018-0189-z)
Supplement: Supplementary file 3 — Additional file 3. Articles included in the categories characteristics of patients, staff attitudes, and outcomes (N = 34). [file 13033_2018_189_MOESM3_ESM.docx]

**Additional file 3**

**Table 2. Articles included in the categories characteristics of patients, staff attitudes, and outcomes (N=34)**

| Characteristics of patients | Local psychiatric beds appear to decrease the use of involuntary admission (Myklebust et al., 2014). | Observational. Retrospective study of case-registries from two Norwegian areas for 2003-2006. 5538 individual treatment episodes were included. | The diagnostic category of psychosis was the strongest predictor of involuntary admission, followed by male gender, and deinstitutionalized service-system. |
| --- | --- | --- | --- |
| Characteristics of patients | Can case-mix explain differences in involuntary admissions to Norwegian psychiatric hospitals? (Bjørngaard & Heggestad, 2001). | Observational. Data from a cross-sectional registration of hospitalized patients on 1 November 1999 from 32 psychiatric hospitals with 1500 patients. | Predictors of involuntary admission (section 5): diagnosis of schizophrenia, acute ward admission, first time admission (negative predictor), etc. Significant predictors of involuntary observation (section 3): age (negative predictor), first time admission, acute ward admission, etc. |
| Characteristics of patients | Acute admissions among immigrants and asylum seekers to a psychiatric hospital in Norway (Iversen & Morken, 2003). | Observational. Hospital records of admissions to one psychiatric hospital (3053) in the period 1995-2000 were retrospectively examined. | The relative risk of admission was similar for immigrants and Norwegians (1.07), while the relative risk for asylum seekers compared to Norwegians was 8.84. Coerced admissions were significantly higher for immigrants. |
| Characteristics of patients | Differences in acute psychiatric admissions between asylum seekers and refugees (Iversen & Morken, 2004). | Observational. Retrospective study of records of 53 asylum seekers and 45 refugees acute admitted to a Norwegian hospital between 1995-2000. | The asylum seekers were more often than the refugees voluntarily admitted (84.9% vs. 57.8%, X^2^=8.97, p<0.005). |
| Characteristics of patients | Narcissism in patients admitted to psychiatric acute wards (Svindseth et al., 2008). | Observational.186 patients were sampled from admission to an acute ward in 2005-2006. Patients were interviewed with the BPRS, GAF and filled out the HADS, the NPI-21, the Rosenberg Self-Esteem Scale. | Being involuntarily admitted was associated with high narcissism scores. Severe violence and high self-esteem were significantly associated with high narcissism. |
| Characteristics of patients | A prospective study of lipids and serotonin as risk markers of violence and self-harm in acute psychiatric patients (Roaldset et al., 2011). | Observational. Prospective study of 256 (of which 46, 18% were involuntary) patients admitted to an acute psychiatric ward in a one-year period (2006-2007). | For the subsample of involuntarily admitted patients, total cholesterol AUC of inpatient violence was 0.67. 14 (82%) of the 17 violent patients during inpatient stay were involuntarily admitted. |
| Characteristics of patients | Patients’ own statements of their future risk for violent an self-harm behaviour (Roaldset et al., 2010). | Observational. Prospective study of 489 (of which 96 were involuntary) patients admitted to an acute psychiatric ward in a one-year period (2006-2007). | Patients’ predictions of own violence, suicidal behaviour, and self-harm were significant. |
| Characteristics of patients | A cross-sectional prospective study of seclusion, restraint and involuntary medication in acute psychiatric wards (Husum et al., 2010). | Observational. Sample of 3572 patients (of which 1214 (35%) were involuntary) from 32 acute wards, study period 2005-2006. | Of the 1214 involuntary patients, 424 (35%) had been secluded, 117 (10%) had been restrained and 113 (9%) had received involuntary depot medication at discharge. There was substantial between-ward variance in the use of coercive measures. |
| Characteristics of patients | Characteristics of psychiatric inpatients who experienced restraint and those who did not (Knutzen et al., 2011). | Retrospective case-control study of restrained patients (375) and random controls (374) from three acute psychiatric wards during a two-year period (2004-2005). | Restrained patients were more likely to be involuntarily referred (X^2^=151.64, df=1, p<0.001). |
| Characteristics of patients | Substance abuse in patients admitted voluntarily and involuntarily to acute psychiatric wards (Opsal et al., 2011) | Observational. Multicenter, cross-sectional, national study, 2005-2006. | 30% (361) of acute patients were involuntarily admitted. Involuntary patients were younger, had less often higher education, more severe symptoms, less often had suicidal ideation/plans, were more often intoxicated, more often had police assistance, more often had a diagnosis of schizophrenia. |
| Characteristics of patients | Increasing mortality in schizophrenia (Høye et al., 2011). | Observational. 1111 patients with schizophrenia admitted to a hospital 1980-2006, linked to the Causes of Death Register. | The women who had always been admitted voluntarily had a higher standardized mortality rate (6.1, 95%CI=3.7-9.9, p=0.006). |
| Characteristics of patients | Clinical differences between immigrants voluntarily and involuntarily admitted to acute psychiatric units (Iversen et al., 2011). | Observational. Immigrants admitted to one of two acute wards 2005-2008. Of 94 in total, 48 were immigrants, 24 refugees and 21 asylum-seekers. Data analysed with Chi-square tests, t-tests and ANOVA. | 66% (62) were voluntary, 31.9 (30) involuntary. Men were more often involuntary and involuntary stays were longer. The involuntary had more often attempted suicide, and these patients were more often unemployed and had less education. The involuntary patients had more symptoms and poorer function at admission. |
| Characteristics of patients | Factors of importance to involuntary admission (Myklebust et al., 2012). | Observational. 1963 admissions to a psychiatric hospital 2003-2006 were analysed. | 9.3% (183) admissions were involuntary. Involuntary admission was predicted by psychosis, emergency admission, and negatively predicted by anxiety. |
| Characteristics of patients | Treatment needs, diagnoses and use of services for acutely admitted psychiatric patients in northwest Russia and northern Norway (Sørgaard et al., 2013). | Observational. Prospective multi-center study of patients admitted acutely to one Russian and two Norwegian hospitals. 841 patients (377 Norwegian and 474 Russian) included. | In Norway, 19.2% were involuntarily observed and 19.2% were involuntarily treated while 33.8% were involuntarily admitted in Russia. Significantly more were voluntary in Russia (Pearson Chi-square 111.02, p=0.000). |
| Characteristics of patients | Predictors of involuntary hospitalizations to acute psychiatry (Hustoft et al., 2013). | Observational. 3326 patients from 20 acute units during a three-month recruitment period in 2005-2006. | 1453 (44%) were referred to either involuntary observation (28%) or involuntary indefinite stay (16%). Some predictors of involuntary admission were: contact with police, referred from someone who without prior knowledge of patient, other contact within last 48 hours. |
| Characteristics of patients | Characteristics of patient frequently subjected to pharmacological and mechanical restraint (Knutzen et al., 2014). | Observational. Retrospective study of records at three acute wards of restrained patients (373), 2004-2005. | Legal basis for stay was not associated with number of restraint episodes among those that had been restrained. |
| Characteristics | Compulsory medical treatment in an emergency psychiatric department (Christensen & Onstad, 2003). | Retrospective case-control study of records at an acute ward 1996-2000. | Of the 340 committed patients, 19% were involuntarily medicated. 69% of these were women. |
| Staff attitudes | Attitudes to coercion among health-care workers and the general public in Norway (Wynn et al., 2006). | Observational. 1094 people filled out an online questionnaire detailing responses to emergency situations. | The mean rate of willingness to use coercion varied from 37.8% (non-violent patient) to 81.1% (violent patient). |
| Staff attitudes | Psychologists and coercion (Wynn et al., 2007). | Observational. 340 psychologists filled out an online questionnaire detailing responses to emergency situations. | The mean rate of willingness to use coercion varied from 30.0% (non-violent patient) to 80.2% (violent patient). |
| Staff attitudes | The staff attitude to coercion scale (Husum et al., 2008). | Observational. Prospective study involving 215 staff from acute and sub-acute wards. | Testing of reliability, validity and feasibility of a rating scale for attitudes to coercion. Three subscales: coercion as offending, coercion as care and security and coercion as treatment. |
| Staff attitudes | Attitudes among stakeholders towards compulsory mental health care in Norway (Diseth et al., 2011). | Observational. 62 respondents (including former patients and relatives of patients, doctors, lawyers) assessed 30 statements on the use of coercion. | The differences in attitude could in part be explained by the respondents’ roles in health care. |
| Staff attitudes | Attitudes to coercion at two Norwegian psychiatric units (Wynn et al., 2011). | Observational. Case-history based questionnaire with responses from 180 staff at two psychiatric units in two hospitals. | Staff were more likely to favour a highly restrictive intervention when the patient was violent. There was little variance in responses from different groups of staff. |
| Staff attitudes | Staff attitudes and thoughts about the use of coercion in acute psychiatric wards (Husum et al., 2011). | Observational.651 staff at 33 acute wards filled out the Staff Attitude to Coercion Scale. Multilevel regression analysis applied. | Most of the variance could be attributed to differences between staff within wards. Most of the staff agreed with the ‘Coercion as care and security attitude’, followed by the ‘Coercion as offending attitude’ and the ‘coercion as treatment attitude’. |
| Staff attitudes | A qualitative exploration of how health care workers in an inpatient setting in Norway experience working with patients who self-injure (Mattson & Binder, 2012). | Observational.8 staff at a secure ward were interviewed about experiences and attitudes to patients who self-injure. Interpretative phenomenological method and systematic text condensation. | Main themes: Before: the frustration inherent in using coercive strategies, The change: from coercion to alliance, Now: the experience of useful ways of working with self-injury, Thin line between life and death. |
| Staff attitudes | Informal coercion in psychiatry (Valenti et al., 2015). | Observational. Focus groups with mental health professionals in 10 countries, examination of attitudes and experiences of informal coercion. | Main themes: a belief that informal coercion is effective, an often uncomfortable feeling using it, an explicit dissonance between attitudes and practice, an implicit dissonance, a link to principles of paternalism and responsibility vs. respect for the patient’s autonomy. |
| Staff attitudes | Terkelsen & Larsen, 2012. | Qualitative study of fieldwork (interviews, observation) involving 22 staff and 12 patients at a locked acute ward. | Staff saw medication as a matter of necessity even when they had to use force and argued that favourable effects made up for side effects. Patients experienced medication as involuntary even when defined as voluntary. |
| Outcomes | A systematic intervention to improve patient information routines and satisfaction in a psychiatric emergency unit (Johnsen et al., 2006). | Intervention study. 433 patients completed questionnaires at one emergency unit. Patients were given standardized information and asked about satisfaction with information, general satisfaction, etc. | Satisfaction with information quality improved as did satisfaction with legal information. General satisfaction with stay did not improve. Patients that had received involuntary medication also experienced an improvement in the quality of information related to this. No differences in general satisfaction scores between voluntary and involuntary patients. |
| Outcomes | Non-adherence to antipsychotic medication, relapse and rehospitalisation in recent-onset schizophrenia (Morken et al., 2008) | Observational. 50 patients with recent onset schizophrenia and related disorders. | Patients that were adherent with oral or depot antipsychotics had fewer days of inpatient coercion (p<0.004). |
| Outcomes | Patient-guided crisis admissions for severe psychotic conditions (Heskestad & Tytlandsvik, 2008). | Intervention study. Two beds were converted into beds utilized by patients without referral. Admissions of 18 patients allowed to use the two beds were examined before and after the change. | The frequency of admissions increased, but the number of bed-days decreased 33% and the number of coerced bed-days was reduced with about 50%. |
| Outcomes | A 2-year follow-up of involuntary admission’s influence upon adherence and outcome in first-episode psychosis (Opjordsmoen et al., 2010). | Observational. Comparison of first admissions of voluntary and involuntary patients at three units. | Female participants were more often involuntary, the involuntary had higher positive and negative PANSS scores and lower GAF symptom and function scores at intake. No difference between the groups after two years. |
| Outcomes | A study of outcome in patients treated at a psychiatric emergency unit (Svindseth et al., 2010) | Observational.147 patients sampled from two emergency wards in 2005-2006. | Negative admission experiences and involuntary admissiondid not influence outcome in terms of BPRS total scores. |
| Outcomes | Hospitalisation of severely mentally ill patients with and without problematic substance use before and during Assertive Community Treatment (Clausen et al., 2016) | Naturalistic observation study including 142 patients from 12 ACT teams. Data from questionnaires and the Norwegian Patient Register. | The number of involuntary inpatient days decreased significantly for patients with problematic substance use after the patients had been included in ACT (mean reduction 55.69, 95%CI=19.16-92.22, p<0.003). |
| Outcomes | Hospitalisation of high and low inpatient service users before and after enrolment into Assertive Community Treatment teams (Clausen et al., 2016). | Naturalistic observation study including 142 patients from 12 ACT teams. Data from questionnaires and the Norwegian Patient Register (two years prior to enrolment in ACT and during ACT). | Of the 142 patients, 74 (52%) were high users of inpatient services before ACT. Number of involuntary inpatient days decreased for the total population, and for high users after the patients had been included in ACT. |
| Outcomes | Bedre tid med brukerstyrte innleggelser (Støvind et al., 2012). | 24 patients were given the opportunity to self-refer to inpatient stays at a District Psychiatric Centre. | The number of involuntary days were reduced to 47 compared to 122 in a similar period preceding the intervention (61% reduction). |
